# Neuron-specific enolase in hypertension patients with acute ischemic stroke and its value forecasting long-term functional outcomes

**DOI:** 10.1186/s12877-023-03986-z

**Published:** 2023-05-15

**Authors:** Lingfei Gao, Jiali Xie, Haiqin Zhang, Hangqi Zheng, Wanjun Zheng, Chunyang Pang, Yunlei Cai, Binbin Deng

**Affiliations:** 1 Department of Neurology, First Affiliated Hospital of Wenzhou Medical University, Wenzhou, China; 2 First Clinical College of Wenzhou Medical University, Wenzhou, China; 3 Department of Neurology, Shanghai East Hospital, Tongji University, Shanghai, PR China; 4 Anyang District Hospital, Dengta Road, Beiguan District, Anyang City, Henan Province PR China

**Keywords:** Neuron specific enolase, Functional outcome, Nomogram, Hypertension, Stroke

## Abstract

**Background:**

Neuron Specific Enolase (NSE), a neuro-biochemical protein marker, may correlate with the prognosis of stroke patients. Moreover, hypertension is the most common comorbidities in patients with acute ischemic stroke (AIS), and the relationship between NSE levels and long-term functional outcomes in such an increasingly large population is unclear. The aim of the study was to investigate the relationships mentioned above and optimize the prediction models.

**Methods:**

From 2018 to 2020, 1086 admissions for AIS were grouped as hypertension and non-hypertension, while hypertension group was randomly divided into development and validation cohorts for internal validation. The severity of the stroke was staged by National Institutes of Health Stroke Scale (NIHSS) score. Stroke prognosis after 1 year of follow up was documented by modified Rankin Scale (mRS) score.

**Results:**

Analysis revealed the following findings:(i) Serum NSE levels increased greatly in hypertension subjects with poor functional outcomes(p = 0.046). However, there was no association in non-hypertension individuals(p = 0.386). (ii) In addition to the conventional factors (age and NIHSS score), NSE (OR:1.241, 95% CI: 1.025–1.502) and prothrombin time were significantly related to the incidence of unfavorable outcomes. (iii)Based on the above four indicators, a novel nomogram was established to predict the prognosis of stoke in hypertension patients with the c-index values of 0.8851.

**Conclusions:**

Overall, high baseline NSE is associated with poor 1-year AIS outcomes in hypertension patients, suggesting NSE may be a potential prognostic and therapeutic target for stroke in hypertension patients.

**Supplementary Information:**

The online version contains supplementary material available at 10.1186/s12877-023-03986-z.

## Background

Stroke is a major cause of disability and death, among which acute ischemic stroke (AIS) accounts for almost 80% [1, 2]. Furthermore, the largest contributor to the global burden of neurological disease is undoubtedly stroke, highlighting a need to improve resources for stroke prevention, management and prognosis globally [3]. Identifying prognostic indicators in AIS enables physicians to confer more timely and valid interventions.

Neuron-specific enolase (NSE), one of the several isoenzymes of enolase, is a glycolytic enzyme. Moreover, NSE has aroused much concern as an auxiliary examination for small-cell lung carcinoma, Creutzfeldt–Jakob disease and neuroendocrine tumors [4]. The enzyme is thought to be released from neuronal and glial tissues into the blood when cell membrane integrity is lost, while the blood-brain barrier is often compromised in stroke patients, the determination of serum NSE level may be a method to predict the prognosis of brain damage [5]. New evidence suggests that high serum NSE concentrations have a high predictive value for early neurobehavioral outcomes after acute stroke [6–8]. However, the predictive ability of patients’ serum NSE levels for long-term outcomes in hypertension patients with stroke was rarely reported.

Hypertension and NSE are often inextricably linked. Previous studies provided preliminary evidence suggesting that raised NSE levels could indicate early brain damage in hypertension patients [9]. In patients with hypertension, increased serum NSE levels were associated with more severe white matter lesions. Moreover, white matter lesions may arise from factors related to brain hypoperfusion and disruption of the blood-brain barrier, leading to decreased cerebral blood flow and thus cerebral ischemia. What’s more, hypertension is undoubtedly the most common co-existing disease and a risk factor for AIS [10]. Compared with patients with normal blood pressure, hypertension patients had poorer stroke outcomes, including an increased incidence of post-stroke death and disability [11, 12]. Due to the increasing and aging population, AIS combined with hypertension has increasingly become a question of concern to scholars, which emphasizes the necessity to evaluate and intervene early in hypertension patients with AIS.

Serum concentrations of NSE have been reported to be as significantly raised in stroke patients compared to controls and to correlate with stroke symptom severity, suggesting that NSE has some clinical predictive potential [13]. Nevertheless, significant conclusions from the above researches are limited owing to variable time points for blood sample collection, heterogeneous populations and the lack of long-term prognosis data. We hereby discuss the relationship between NSE and the long-term prognosis of AIS in hypertension patients, explore the incremental predictive ability of baseline NSE and add it to the long-term nomogram of AIS prognosis for hypertension patients.

In this study, we aimed to investigate 1): the association between high NSE serum levels and long-term AIS prognosis in hypertension patients, and 2): the incremental predictive ability of serum NSE levels adding to the conventional model. The new nomogram constructed in this study may facilitate the prediction of poor prognosis in hypertensive stroke patients.

## Materials and methods

### Study participants

This study was approved by the Ethics Committee of the First Affiliated Hospital of Wenzhou Medical University and conformed to the Helsinki Declaration. From 2018 to 2020, we had 1086 admissions for AIS. Of these, 933 were hypertension patients and 153 were non-hypertension patients. Exclusion criteria: 1, excluding other vascular infarction, cerebral venous thrombosis; 2, patients with the transient ischemic attack, cerebral hemorrhage or subarachnoid hemorrhage;3, lack of outcome variables after 1-year follow-up;4, patients with peripheral vascular diseases, central nervous system disorders, and neuroendocrine cell-derived hyperplasia or tumors. All patients were admitted within 7 days of stroke onset, and their demographic, clinical characteristics, past medical history, and imaging findings were collected using standardized data records. Finally, 457 hypertension and 142 non-hypertension patients with AIS meeting the inclusion criteria were included as the hypertension group and non-hypertension group, respectively.

Referring to previous research findings [14–16], two-thirds of the hypertensive patients (n = 304) were randomly selected as the development cohort and one-third (n = 153) as the validation cohort in R (package caret).

## Clinical and laboratory assessments

All patients underwent CT examination within 24 h of admission, but the final diagnosis was confirmed by repeat CT and/or MRI performed on the third and seventh days after admission. Hypertension was defined according to (i) confirmed medical history or (ii) new diagnosis during hospitalization based on clinical or laboratory examination: systolic blood pressure ≥ 130 mmHg or diastolic blood pressure ≥ 80 mmHg on repeated measurements for diagnosis of hypertension [17]. All blood indicators were tested after overnight fasting the morning after admission. After excluding variables with too many missing values, we included 36 variables for analysis.

## Patient assessment and follow-up

National Institutes of Health Stroke Scale (NIHSS) scores were assessed on admission for all patients, and functional outcomes were further assessed after 1 year using a modified Rankin Scale (mRS) score [18] by telephone consultations, questionnaire surveys or outpatient reviews. Good outcomes were defined as mRS scores of 0–2, while poor outcomes were defined as mRS scores of 3–6.

### Statistical analysis

Patient baseline data and risk factors were statistically analyzed by SPSS. Categorical variables were compared with the chi-square test, and continuous variables were compared with the nonparametric test or t-test. A multivariable logistics regression model was performed to identify independent predictors. Odds ratios (ORs) with their 95% confidence interval (CI) were reported. After multivariable logistic regression analysis and calculation of risk factors, three models were constructed and the receiver operating characteristic (ROC) curves, the integrated discrimination improvement (IDI), the net reclassification index (NRI) values were calculated to evaluate the incremental prognostic value of NSE levels beyond conventional risk factors. The nomogram and calibration curves were performed with package rms in the R version. P < 0.05 was considered statistically significant and all calculations were based on SPSS version 22.0 software and R.

## Results

### Baseline characteristics and study outcomes

**Fig. 1:**
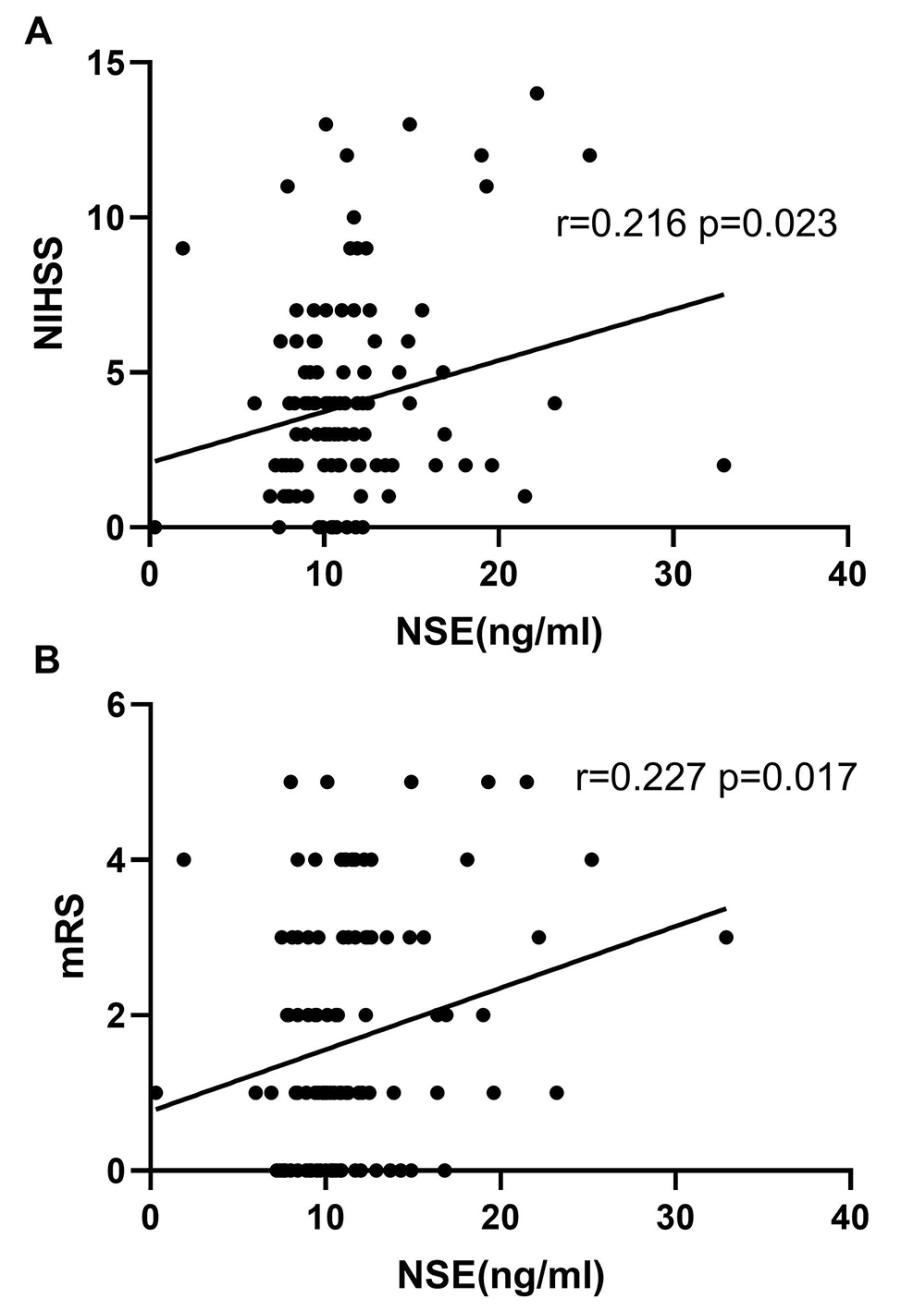
Correlation between NSE and clinical outcomes (mRS and NIHSS score) of hypertension patients. Abbreviation: NSE: Neuron specific enolase; mRS: modified Rankin Scale; NIHSS: National Institutes of Health Stroke Scale

The general baseline data of patients is shown in Table 1. A total of 457 hypertension patients were included in this study, with 304 randomized as the development cohort and 153 as the validation cohort. Of all the patients with hypertension in the development cohort, 227 (74.7%) participants experienced good outcomes (mRS ≤ 2) and 77 (25.3%) participants developed poor outcomes (mRS > 2). Patients attributable to good outcomes had an average age of 64.3 years and an average of NIHSS of 2.9, with 62.6% male predominance; in the group with poor outcomes, the average age was 70.2 years and the average NIHSS was 6.6, with 68.8% male predominance. Between the two groups, NSE was statistically significant but the associated biomarkers were not. Moreover, a significant correlation between NSE serum levels with NIHSS scores (r = 0.216 and p = 0.023) and with mRS scores (r = 0.227 and p = 0.017) in AIS was shown in Pearson correlation test (Fig. 1), where the relationship between them exhibited a positive correlation with a strong correlation power. In general, high NSE levels were associated with severity at admission and poor prognosis at 1 year.

### Correlation of serum NSE level with 1-year functional outcomes

**Fig. 2:**
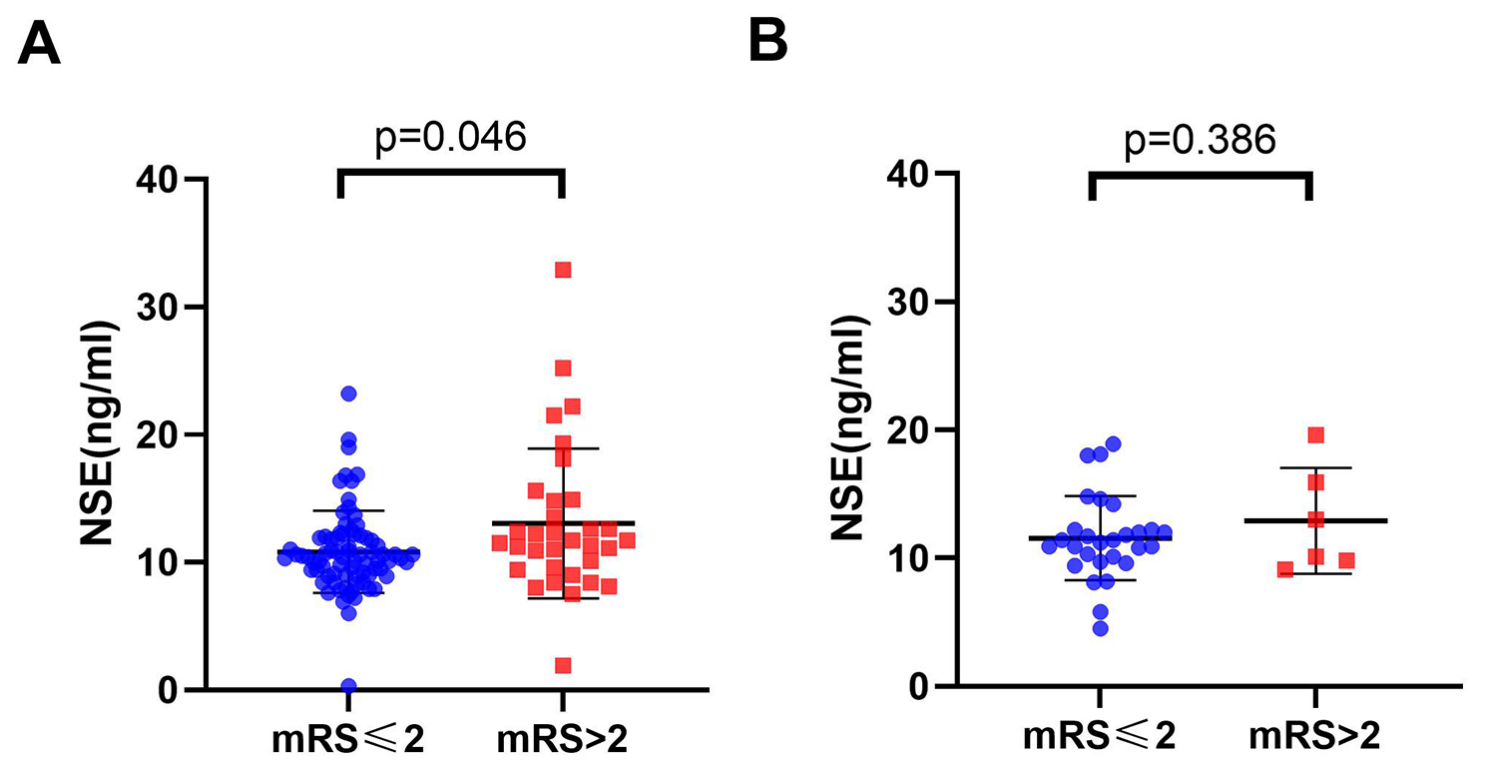
The distribution of NSE with different mRS groups. **A:** hypertension group; **B:** non-hypertension group. The scatter diagram in the distribution of NSE between the mRS ≤ 2 and mRS > 2 groups, respectively. Abbreviation: NSE: Neuron specific enolase; mRS: modified Rankin Scale

**Fig. 3:**
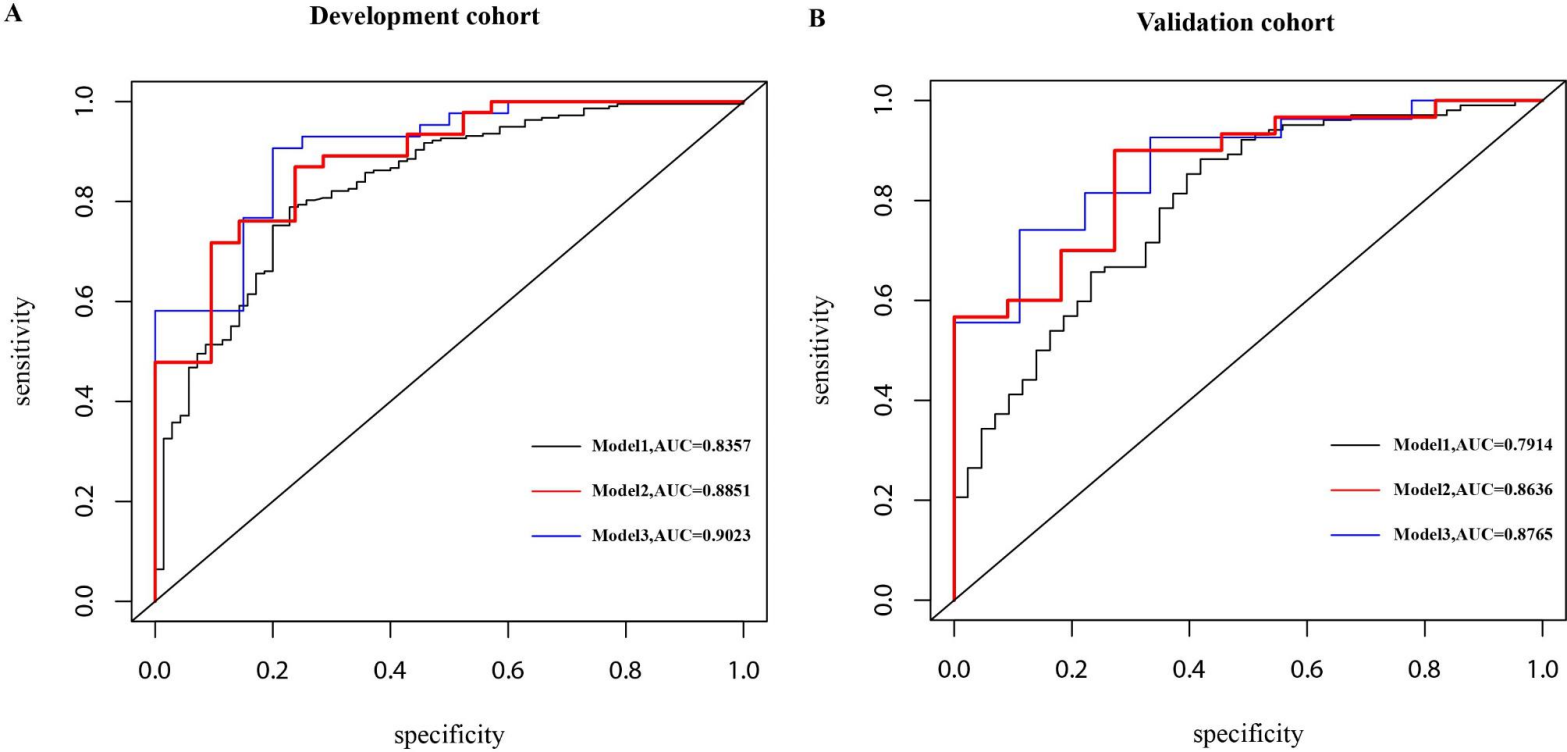
The ROC curves of the three models. model 1: NIHSS + Age + PT, AUC = 0.8357; model 2: NIHSS + Age + PT + NSE, AUC = 0.8851; model 3: NIHSS + Age + PT + NSE + HbA1C, AUC = 0.9023. We compare the three models: In the development cohort: model 1vs model 2 :z = − 1.838, p = 0.066;model2 vs. model3:z = 0.814,p = 0.420; In the validation cohort: model 1 vs. model 2: z = 0.329, p = 0.740; model2 vs. model3: z = 0.632,p = 0.527. Abbreviation: ROC: receiver operating characteristic; AUC: Area Under Curve

Univariate and multivariate analyses were used to identify potential prognostic factors in hypertension patients, and we used logistic regression to this end. In the univariate analysis, NSE was statistically different (p = 0.046) in the stroke prognosis of the hypertension group (Fig. 2A). However, in the non-hypertension group, NSE was not significant (p = 0.386) (Fig. 2B). Through multivariate analysis, we considered the age of patients [odds ratio (OR): 1.164, 95% CI: 1.046–1.296], the NIHSS on admission (OR: 1.670, 95% CI: 1.050–2.656), prothrombin time (PT) (OR: 0.069, 95% CI: 0.008–0.597), and NSE (OR:1.241, 95% CI: 1.025–1.502), a total of four variables (p < 0.05) as significant factors on the unfavorable functional outcome as evaluated by the mRS > 2 at 1 year in the development cohort (Table 2). Moreover, the variable NSE (OR: 1.153, 95% CI: 1.016–1.309) demonstrated statistical significance during multivariate analysis of the total cohort (Supplementary Table 1). In order to verify whether NSE would improve the capacity to predict stroke prognosis, two additional prediction models based on NSE have been constructed: model 1: age + NIHSS + PT and model 2: age + NIHSS + PT + NSE. Baseline factors with p < 0.1, constitutive of age, the NIHSS, PT, NSE and HbA1C in multivariate analysis (Table 2) were obtained in model 3. ROC curves were shown in Fig. 3, and the three AUCs were 0.8357, 0.8851 and 0.9023, respectively. In the validation group, we discovered that the AUCs of the three ROCs reached 0.7914, 0.8636, and 0.8765, respectively.

**Table 1:** Demographic and clinical characteristics

	Total				Development cohort			
	Good	Poor	P-value		Good	Poor	P-value	
**N**	329	124			227	77		
**Demographic data**								
Age	64.64 ± 11.34	70.07 ± 9.86	< 0.001*		64.33 ± 11.74	70.21 ± 9.05	< 0.001*	
Sex(male,n)	206(61.9%)	82(66.0%)	0.401		142(62.5%)	53(68.7%)	0.321	
SBP	163.20 ± 17.46	161.48 ± 17.43	0.347		163.56 ± 18.16	160.09 ± 16.08	0.138	
DBP	85.07 ± 13.28	83.60 ± 122.41	0.285		85.78 ± 14.19	82.45 ± 10.80	0.033*	
Admission NIHSS scores	2.86 ± 2.16	6.47 ± 4.06	< 0.001*		2.92 ± 2.24	6.61 ± 3.95	< 0.001*	
BMI	24.86 ± 3.39	24.20 ± 2.95	0.508		25.18 ± 3.75	24.55 ± 3.41	0.634	
**Comorbidities**								
Smoking history(n)	138(41.3%)	52(41.8%)	0.924		95(41.8%)	36(46.7%)	0.453	
Drinking history(n)	115(34.4%)	39(31.4%)	0.535		83(36.6%)	27(35.0%)	0.794	
Diabetes(n)	134(40.4%)	48(38.6%)	0.731		88(38.8%)	24(31.1%)	0.223	
Atrial fibrillation(n)	22(6.5%)	17(13.6%)	0.016*		13(5.6%)	10(12%)	0.037*	
Hyperlipidemia(n)	190(57.2%)	54(44.3%)	0.014*		138(61.1%)	32(41.6%)	0.003*	
**Laboratory findings** **Blood routine**								
Neutrophil	4.13 ± 1.74	5.17 ± 5.08	0.028*		4.08 ± 1.58	5.20 ± 6.15	0.117	
Lymphocyte	1.87 ± 0.64	1.62 ± 0.64	< 0.001*		1.87 ± 0.65	1.61 ± 0.61	0.002*	
NLR	2.64 ± 2.68	3.80 ± 4.03	0.004*		2.51 ± 1.67	3.79 ± 4.42	0.016*	
Platelet	224.75 ± 63.32	221.67 ± 74.05	0.660		223.30 ± 63.23	219.94 ± 71.57	0.697	
**Liver and kidney**								
AST	24.95 ± 14.69	27.65 ± 14.27	0.080		24.55 ± 10.26	26.40 ± 11.37	0.184	
ALT	23.37 ± 14.92	24.75 ± 34.51	0.552		23.73 ± 16.43	20.99 ± 12.76	0.184	
TB	11.80 ± 6.00	12.72 ± 6.43	0.162		11.47 ± 6.73	12.34 ± 6.32	0.320	
Albumin	38.69 ± 3.82	36.89 ± 3.87	< 0.001*		38.78 ± 4.01	36.47 ± 3.98	< 0.001*	
TG	1.85 ± 0.98	2.65 ± 12.19	0.473		1.84 ± 0.95	3.26 ± 15.34	0.417	
TC	4.70 ± 1.17	4.52 ± 1.15	0.148		4.72 ± 1.19	4.48 ± 1.17	0.126	
HDL	1.09 ± 0.29	1.12 ± 0.32	0.330		1.09 ± 0.33	1.09 ± 0.32	0.919	
LDL	2.68 ± 0.84	2.64 ± 0.82	0.635		2.69 ± 0.84	2.62 ± 0.82	0.526	
HCY	10.94 ± 8.64	11.55 ± 9.46	0.574		10.94 ± 9.17	11.44 ± 7.82	0.711	
BUN	5.79 ± 12.99	5.47 ± 2.17	0.786		6.08 ± 15.71	5.17 ± 1.90	0.614	
CR	72.76 ± 29.82	73.31 ± 25.33	0.854		72.54 ± 32.00	73.74 ± 23.97	0.763	
**Thyroid and coagulation**								
TH	105.41 ± 19.74	107.29 ± 19.23	0.374		105.46 ± 20.73	108.31 ± 19.41	0.297	
TSH	1.92 ± 1.23	2.43 ± 5.90	0.358		1.96 ± 1.28	2.72 ± 7.36	0.382	
HbA1C	6.70 ± 1.62	6.26 ± 1.34	0.010*		6.56 ± 1.49	6.08 ± 1.10	0.007*	
PT	13.61 ± 1.84	14.05 ± 1.84	0.027*		13.55 ± 0.75	13.88 ± 0.98	0.004*	
APTT	36.93 ± 4.79	38.04 ± 4.17	0.028*		36.71 ± 4.49	38.35 ± 3.68	0.005*	
D-dimer	1.42 ± 2.39	2.68 ± 5.11	0.061		1.34 ± 1.63	1.58 ± 2.76	0.480	
**Tumor markers within 24 h**								
NSE	10.83 ± 3.22	13.06 ± 5.87	0.046*		10.55 ± 3.40	14.55 ± 6.37	0.010*	
CA125	9.72 ± 8.64	13.85 ± 20.22	0.092		9.88 ± 10.08	11.47 ± 9.22	0.332	
CA153	8.47 ± 4.98	8.59 ± 5.00	0.845		8.40 ± 4.95	8.52 ± 5.16	0.889	
CA199	12.51 ± 26.27	12.43 ± 12.41	0.975		13.30 ± 30.95	10.75 ± 10.37	0.514	

Abbreviation: BMI: Body Mass Index; SBP: Systolic blood pressure; DBP: Diastolic blood pressure; NIHSS: National Institutes of Health Stroke Scale ;NLR :Neutrophil-to-Lymphocyte ratio; AST: Aspartate aminotransferase; ALT: Alanine aminotransferase; TB: Total bilirubin; TG: Triglyceride; TC: Total cholesterol; HDL: High density lipoprotein; LDL: Low density lipoprotein; HCY: Homocysteine; BUN: Blood urea nitrogen; CR: Creatinine; TH: Thyroid hormones; TSH :Thyroid stimulating hormone; PT: Prothrombin time; APTT: Activated partial thromboplastin time ; NSE: Neuron specific enolase. *p < 0.05.

**Table 2:** Multivariate logistic regression according to the functional outcomes

	OR	95%CI	P
Age	1.164	1.046–1.296	0.005*
NIHSS	1.670	1.050–2.656	0.030*
NLR	1.182	0.940–1.486	0.152
Albumin	0.845	0.627–1.139	0.269
Hb1C	0.581	0.306–1.105	0.098
PT	0.069	0.008–0.597	0.015*
APTT	0.912	0.696–1.195	0.504
NSE	1.241	1.025–1.502	0.027*

NIHSS:National Institutes of Health Stroke Scale on admission; NLR :Neutrophil-to-Lymphocyte ratio; PT: Prothrombin time; APTT: Activated partial thromboplastin time; NSE: Neuron specific enolase.*p < 0.05

## Value-added effects of NSE in prognostic models of functional outcomes

**Fig. 4:**
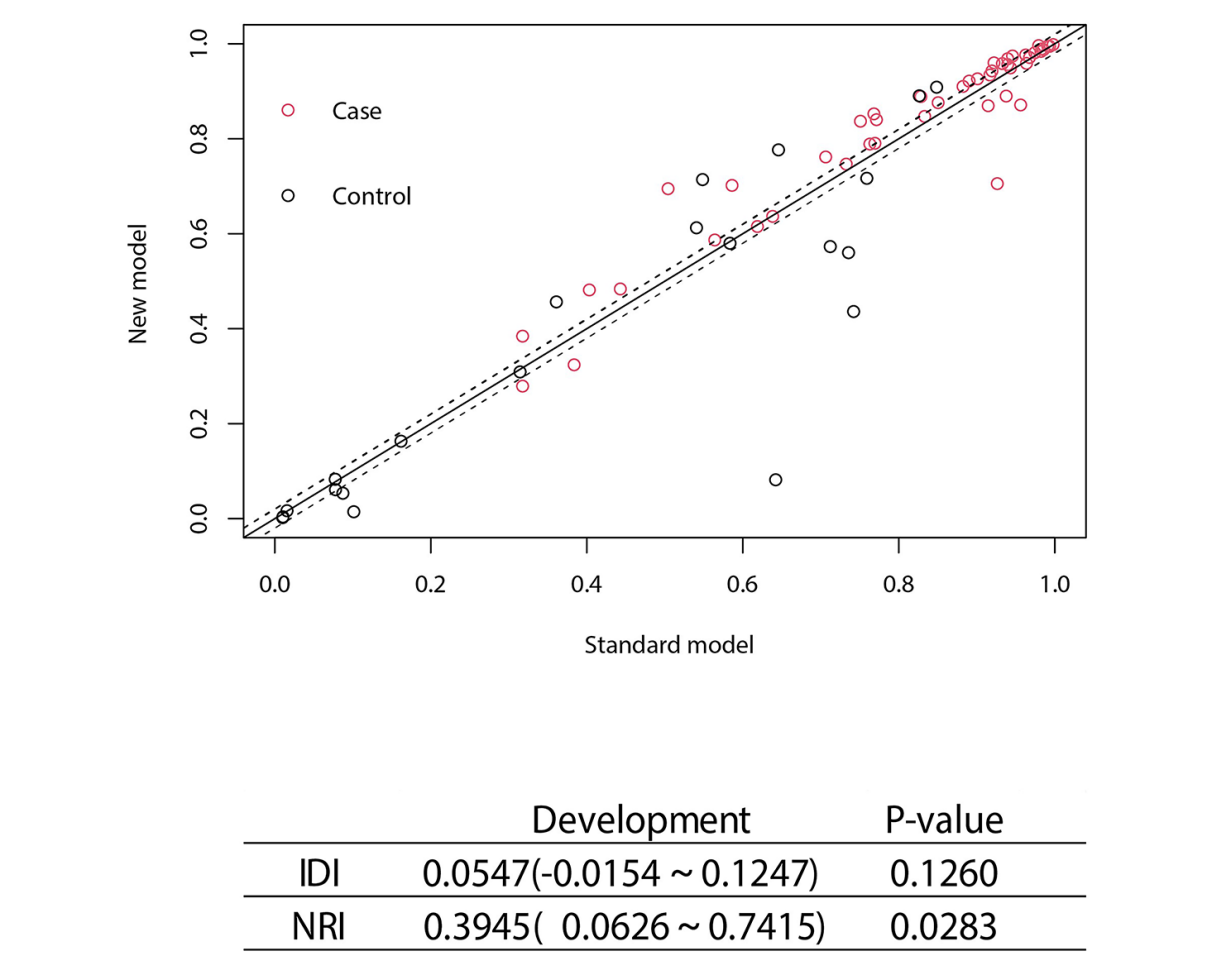
The NRI and IDI between model1 and model2 of the development cohort. Both NRI and IDI show that model2 has a higher predictive power than model1. Abbreviation: NRI: Net reclassification index; IDI: Integrated Discrimination Improvement; NSE: Neuron specific enolase

The difference in the AUCs was indiscriminate between model 2 and model 3 in the development cohort (Z = 0.814, p-value = 0.42) as well as in the validation cohort (Z = 0.632, p-value = 0.53). In order to compare the differences in predictive performance between model 1 and model 2, we calculated NRIs and IDIs. As shown in Fig. 4, the addition of NSE to the model1 improved risk prediction of 1-year AIS outcomes, leading to a weakly significant increase in C-statistics but significant improvement in reclassification (Δ AUC = 0.049, p = 0.066; NRI = 39.45%, p = 0.028; IDI = 5.47%, p = 0.126). As above, model3 had no special optimization capabilities compared to model2(NRI = 30.34%, p = 0.0949; IDI = 3.15%, p = 0.1323).

## A novel prognostic model

**Fig. 5:**
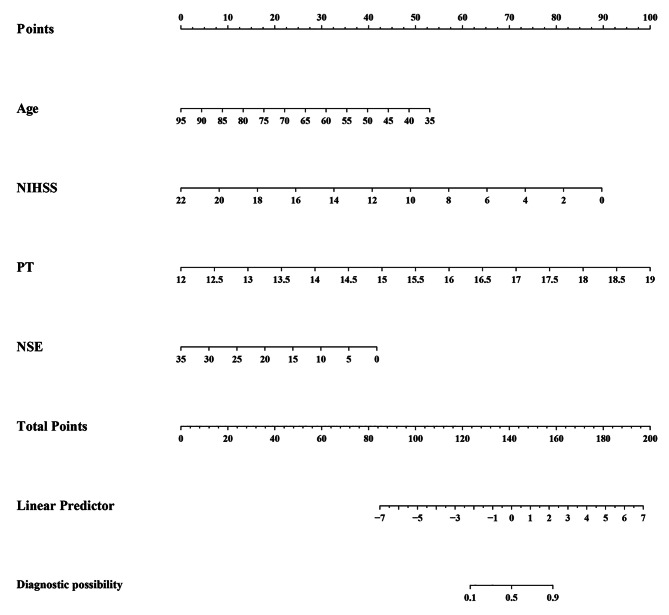
The nomogram for patients with hypertension AIS. To use the nomogram, an individual patient’s value is located on each variable axis, and a line is drawn upward to determine the number of points received for each variable value. The sum of these numbers is located on the Total Points axis, and a line is drawn downward to the survival axes to determine the likelihood of poor outcome

**Fig. 6:**
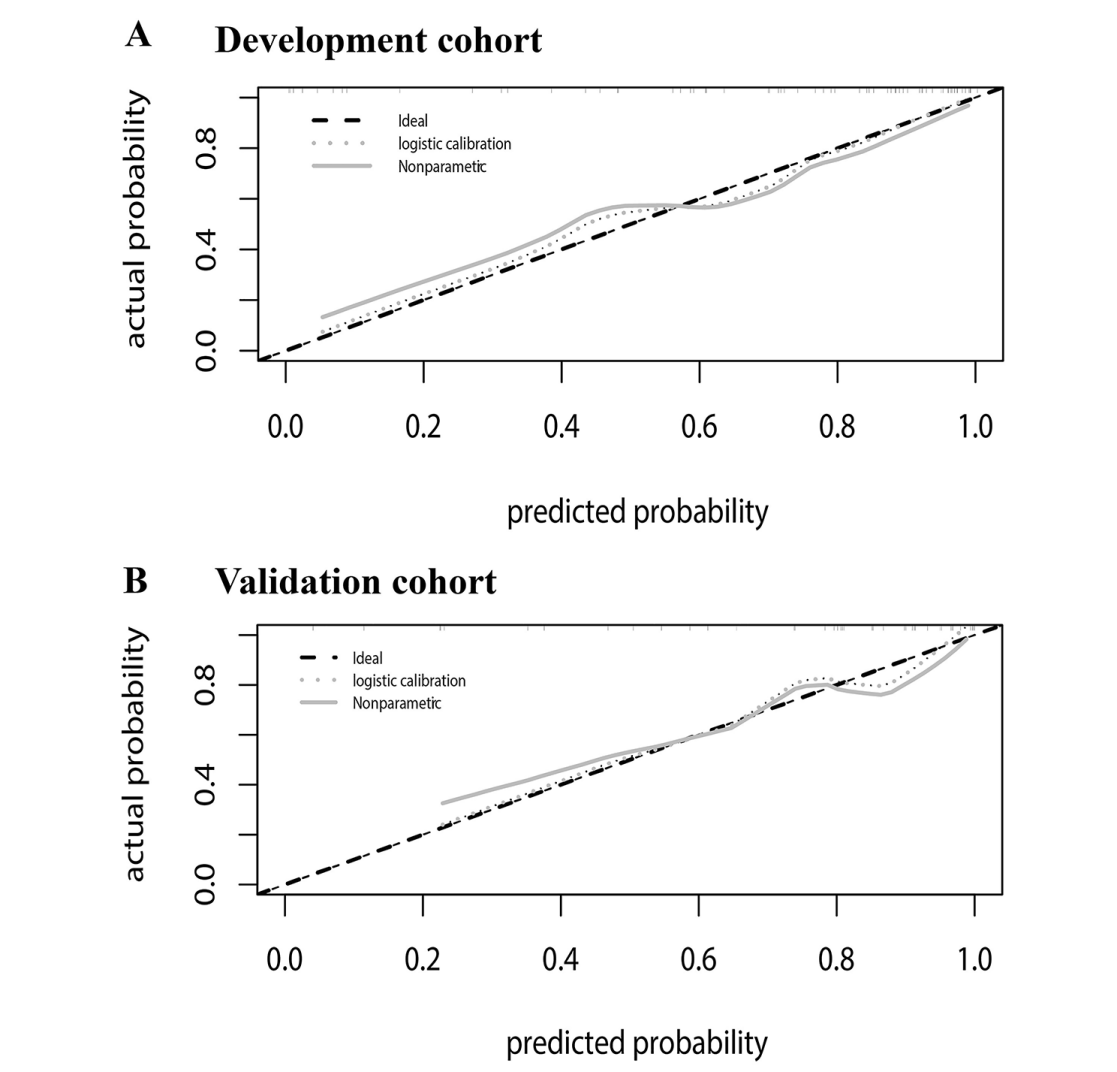
Calibration of the nomogram. Note. the total hypertension group has been randomly divided into development and validation cohorts, with 2 thirds in the development and 1 third in the validation

Clearly, we selected model 2 to establish a nomogram for hypertension patients to predict an unfavorable outcome (Fig. 5). The best agreement between nomogram predictions and actual observations was presented in prognostic calibration plots of nomogram (Fig. 6). In both the modeling and validation cohort, the calibration plot matched the actual conditions almost perfectly. In the validation cohort, the AUC of Model2 reached 0.8636, which further illustrated the excellent predictive ability of the model.

## Subgroup analysis of NSE and functional outcome

**Fig. 7:**
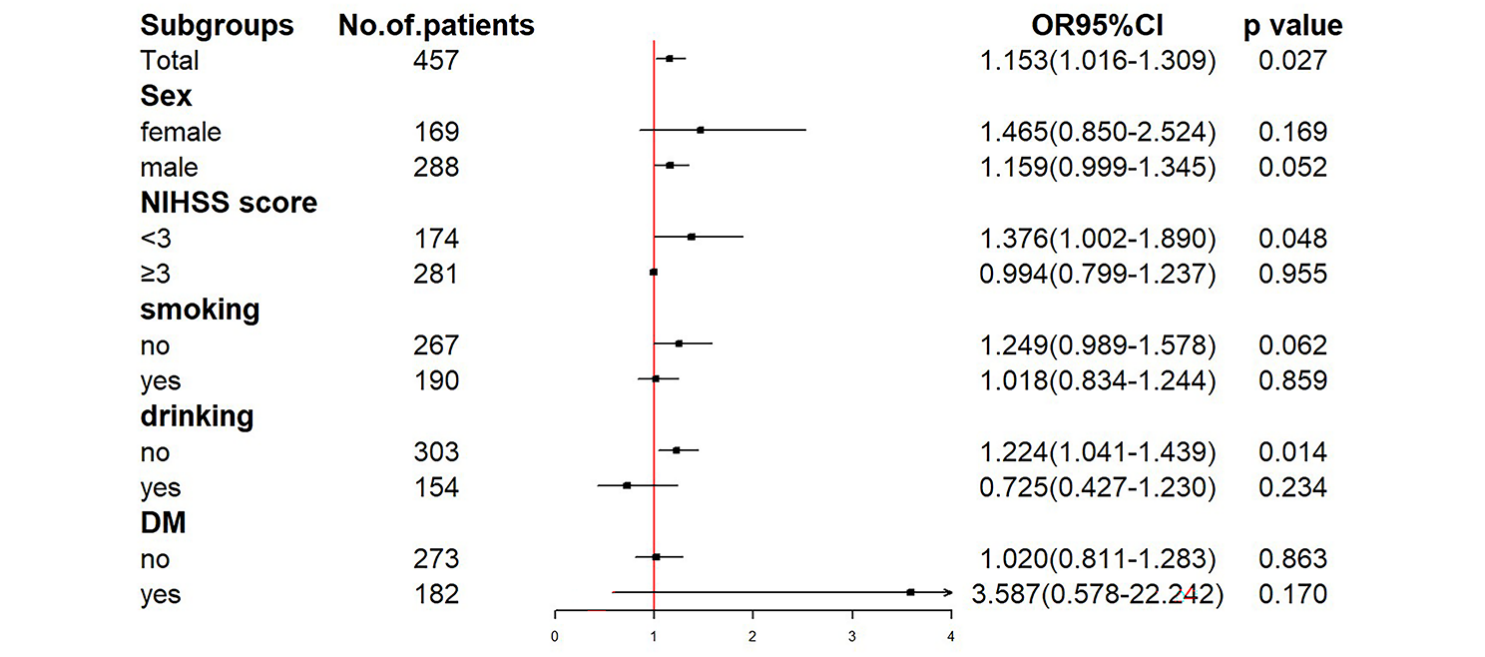
Forest plot of subgroup analysis for the association between NSE and functional outcome at 1 year. Abbreviation: NIHSS: National Institutes of Health Stroke Scale; DM: diabetes mellitus

The study included a subgroup analysis stratified by gender, NIHSS on admission, cigarette smoking and alcohol consumption, and history of diabetes mellitus, to investigate the correlation between NSE and individuals’ prognosis (mRS). The results indicated that a pattern difference between the NIHSS strata was observed in the adjusted relationships between NSE and 1-year outcomes (Fig. 7). This finding suggests that the prognostic value of NSE for 1-year AIS outcomes was more prominent in groups with the NIHSS < 3.

## Discussion

Using the developmental cohort of the forward-looking and continuous hospital stroke registry at the First Affiliated Hospital of Wenzhou Medical University, we have established a nomogram based on age, the NIHSS, PT and serum NSE levels, which was constructed to explore the underlying influence of NSE in the prediction of functional outcomes. While high NSE serum levels in the acute phase were associated with poor outcomes in stroke patients were confirmed in previous research [19], this is the first study to explore the relationship between serum NSE levels and long-term prognosis of acute ischemic stroke in hypertension patients.

The mechanism of the interaction between serum NSE levels and stroke is still being explored. NSE, the most acidic brain isoenzyme of glycolytic enzyme enolase, is present in the cytoplasm of neurons and neuroendocrine differentiated cells and may play a dual role in neuroinflammation and neuroprotection in some nervous system events. The metabolic syndrome (including hypertension, dyslipidemia, insulin resistance and obesity) had been shown to lead to a chronic pro-inflammatory state and a continuous circulation of cytokines in the body, especially tumor necrosis factor-alpha (TNF-α). This syndrome can cause endothelial cell dysfunction and increased blood-brain barrier permeability [20]. On the one hand, large amounts of cytokine-rich plasma (including TNF-α cytokines) leach through the damaged blood-brain barrier [21]. TNF-α can induce increased glutamate release, leading to excitatory toxicity and disruption of neuronal blood flow. In the meantime, a series of physiological changes caused by hypoxia, such as lipid peroxidation, mitochondrial dysfunction and energy metabolism dysfunction, can trigger neuronal apoptosis. On the other hand, hypertension affects the small arteries in the brain, causing disruption of metabolic processes in astrocytes and nearby neurons [22]. These all lead to the destruction of neurons and the entry of NSE into the bloodstream.

Stroke, in essence, also leads to endothelial cell death and damage to the brain-blood barrier due to short or long periods of hypoxia and ischemia, and the cytosolic content released from injured brain tissue can cross the blood-brain barrier [23]. In the study of Haque et al., it was confirmed that the increase of serum NSE level can increase extracellular matrix degradation, inflammatory glial cell proliferation and actin remodeling, thus affecting the migration of activated macrophages and microglia to the injured area and causing more neuronal cell death [24].

Notably, NSE may also exhibit neurotrophic functions as it can control neuronal survival, differentiation and axonal regeneration through activation of the phosphatidylinositol-4,5-bisphosphate 3-kinase (PI3K) and mitogen-activated protein kinase (MAPK) signaling pathways [24–27]. Moreover, NSE has neurotrophic properties in a wide range of central nervous system neurons and is essential for the survival of neuronal cells [28–30].

In addition, a significant correlation between serum NSE level and NIHSS score in ischemic stroke (r = 0.216, p < 0.023) was found in this study, which is similar to Nasution et al. [19]. The results indicated that the serum NSE level in AIS patients was positively correlated with the severity of the disease. Serum NSE level was also significantly correlated with mRS Score in patients with ischemic stroke. Previous studies reported the relationship between NSE levels and functional prognostic outcomes. Zaheer et al. (2013) obtained the results: There was a positive correlation between NSE levels on day 1 and functional neurological outcomes assessed by mRS on day 30 (r = 0.744, P < 0.001). This is similar to our study, except that we complemented it by examining the relationship between NSE levels and long-term mRS, and confirmed that NSE levels were positively correlated with 1-year MRS in AIS patients (r = 0.227, p = 0.017).

The current study had several limitations. First, the examination of serum NSE levels is taken once, considering the dynamic changes in hormone levels, assessment of hormone levels at multiple time points better reflects the status of NSE levels over time than the assessment of only one-time point. Second, this study uses internal validation and lacks external validation. Future studies should be involved in the external validation method of multicenter cooperation. Third, although we investigated the relationship between serum NSE levels and AIS, we did not consider any biomarker other than NSE (such as the s100b protein, of which levels could predict brain damage).

## Conclusions

Conclusively, high NSE at baseline is associated with poor 1-year AIS outcomes in hypertension patients, especially in patients with NIHSS score less than 3, suggesting that NSE may be a potential prognostic biomarker and therapeutic target for stroke patients with hypertension.

## Electronic supplementary material

Below is the link to the electronic supplementary material.

Supplementary Table 1. Multivariate logistic regression of the total cohort

## Data Availability

All data generated or analysed during this study are included in this published article.

## List of abbreviations

NSE: Neuron Specific Enolase
AIS: acute ischemic stroke
NIHSS score: National Institutes of Health Stroke Scale score
mRS score: modified Rankin Scale score.
