# Supplementary material for: Neuron-specific enolase in hypertension patients with acute ischemic stroke and its value forecasting long-term functional outcomes
Source: BMC Geriatr. 2023 May 15;23:294. doi: 10.1186/s12877-023-03986-z (PMC10184372; doi:10.1186/s12877-023-03986-z)
Supplement: Supplementary file 1 — Supplementary Table 1. Multivariate logistic regression of the total cohort [file 12877_2023_3986_MOESM1_ESM.docx]

| **Supplementary Table1. Multivariate logistic regression of the total cohort** | | | |
| --- | --- | --- | --- |
|  | **OR** | **95%CI** | **P** |
| Age | 1.093 | 1.031-1.158 | 0.003* |
| NIHSS | 1.429 | 1.150-1.777 | 0.001* |
| NLR | 0.954 | 0.868-1.048 | 0.325 |
| Albumin | 1.026 | 0.868-1.213 | 0.761 |
| Hb1C | 0.682 | 0.443-1.049 | 0.082 |
| PT | 0.984 | 0.743-1.304 | 0.912 |
| APTT | 0.955 | 0.808-1.129 | 0.589 |
| NSE | 1.153 | 1.016-1.309 | 0.027* |

NIHSS: National Institutes of Health Stroke Scale on admission; NLR :Neutrophil-to-Lymphocyte ratio; PT: Prothrombin time; APTT: Activated partial thromboplastin time; NSE: Neuron specific enolase.*p<0.05.
